# Overcoming Stagnation in the Levels and Distribution of Child Mortality: The Case of the Philippines

**DOI:** 10.1371/journal.pone.0139458

**Published:** 2015-10-02

**Authors:** Raoul Bermejo, Sonja Firth, Andrew Hodge, Eliana Jimenez-Soto, Willibald Zeck

**Affiliations:** 1 UNICEF Philippines, Makati City, Philippines; 2 Institute of Tropical Medicine, Antwerp, Belgium; 3 School of Public Health, The University of Queensland, Brisbane, Queensland, Australia; 4 Department of Obstetrics and Gynecology, Medical University of Graz, Graz, Austria; Centre Hospitalier Universitaire Vaudois, FRANCE

## Abstract

**Background:**

Health-related within-country inequalities continue to be a matter of great interest and concern to both policy makers and researchers. This study aims to assess the level and the distribution of child mortality outcomes in the Philippines across geographical and socioeconomic indicators.

**Methodology:**

Data on 159,130 children ever borne were analysed from five waves of the Philippine Demographic and Health Survey. Direct estimation was used to construct under-five and neonatal mortality rates for the period 1980–2013. Rate differences and ratios, and where possible, slope and relative indices of inequality were calculated to measure disparities on absolute and relative scales. Stratification was undertaken by levels of rural/urban location, island groups and household wealth.

**Findings:**

National under-five and neonatal mortality rates have shown considerable albeit differential reductions since 1980. Recently released data suggests that neonatal mortality has declined following a period of stagnation. Declines in under-five mortality have been accompanied by decreases in wealth and geography-related absolute inequalities. However, relative inequalities for the same markers have remained stable over time. For neonates, mixed evidence suggests that absolute and relative inequalities have remained stable or may have risen.

**Conclusion:**

In addition to continued reductions in under-five mortality, new data suggests that the Philippines have achieved success in addressing the commonly observed stagnated trend in neonatal mortality. This success has been driven by economic improvement since 2006 as well as efforts to implement a nationwide universal health care campaign. Yet, such patterns, nonetheless, accorded with persistent inequalities, particularly on a relative scale. A continued focus on addressing universal coverage, the influence of decentralisation and armed conflict, and issues along the continuum of care is advocated.

## Introduction

Since the early 1990s the global declines in child mortality have been considerable [1]. Notwithstanding this significant progress, the rate of neonatal mortality has declined at a slower pace than the rate of deaths of children under five [2,3]. The maternal mortality ratio (MMR) remains high in the Philippines, with Millennium Development Goal (MDG) 5 unlikely to be achieved by 2015 [4]. The maternal mortality rate might be strongly correlated with neonatal mortality through various factors, including the continuum of care [5]. In Asia, the burden of preventable child deaths remains high, accounting for approximately 40% of the global burden [1]. Large disparities across populations also persist within individual countries [6–8], including the Philippines [9]. The source of the disadvantage is often linked to socioeconomic status [10,11], however, geography has also been observed as an important marker [6]. Underlying these patterns are often issues related to poor capacity, governance and quality of care [6,9,12]. Consideration of inequalities in access to health services and health outcomes continues to attract the attention of both scholars and policy makers in the public health arena [13,14].

The Philippines is a lower middle-income country that has achieved significant economic and development outcomes in the recent decade [15]. Yet, national averages mask significant disparities. For instance, the annual family income in the National Capital Region (i.e. the location of Manila) averages US$6,058, more than three times the average annual family income observed in the poorest regions [16]. Common childhood illnesses are still prevalent with pneumonia and diarrhoea remaining the leading causes of death for children [17]. Skilled birth attendance and antenatal care coverage has increased across the nation; however, quality of care is inadequate, particularly for women with a lower socioeconomic status [18]. The total fertility rate in the country has reduced from 3.7 in 1998 to 3 in 2013, although it also shows high variability, ranging from 2.3 to 4.2 across regions [18]. Due to concerns related to existing health inequities, the government has adopted an ambitious universal health coverage programme through the Philippine Health Insurance Corporation (PhilHealth), which supports free enrolment for the lowest wealth quintile of the population. This is supported by other range of government programmes, including cash transfers, which have targeted poverty. [19] However, limited evidence exists not only on the impact those programmes have had, but also on the underlying health inequity trends.

This paper presents trends and inequalities of the under-five mortality rate (U5MR) and neonatal mortality rate (NMR). We analysed both absolute and relative inequalities for U5MR and NMR across wealth and geography (urban/rural and island groups). Our analysis updates a previous study [9] which focused on mortality trends and concentration indices of wealth-related inequalities. In comparison, utilising the recently released 2013 wave of the Philippines Demographic and Health Survey (DHS) this study covers a longer time frame (1980–2013 vs 1990–2007) and in line with the most recent literature [8,20,21] examines both absolute and relative measures of inequality. It is well-known that the extent to which inequalities show upward or downward trends over time is strongly influenced by the choice of absolute or relative measures.[22] This study will then allow us to provide more robust conclusions regarding the extent of inequality in the country.

## Methods

### Ethics statement

The datasets used in this study were obtained from the MEASURE DHS website, <http://www.dhsprogram.com>. Full review of this study from an institutional review board was not sought as this manuscript involved secondary data analysis of datasets that are publicly available, anonymous, with no identifiable information on the survey participants. The data can be obtained directly from MEASURE DHS.

### Data

The analyses are based on survey data from the Philippine Demographic and Health Survey (DHS), undertaken by Philippine Statistics Authority together with the worldwide DHS program. The DHS are repeated cross-sectional surveys designed to collect socio-demographic and health data, including information on fertility, family planning, and health and mortality variables. The surveys are representative at the national, urban-rural and regional levels, except the first wave which was not representative of the then recently formed Autonomous Region in Muslim Mindanao. The surveys followed multi-stage sampling designs (two-stage for 1993, 1998 and 2013 and three-stage for 2003 and 2008), which included stratification by region and urban/rural location, and clustering by local settlements called barangays (the primary sampling unit). Further details on data collection, sample design and management procedures are described elsewhere.[18,23–26] We utilised five survey waves (fieldwork months): 1993 (March-May), 1998 (February-April), 2003 (June-September), 2008 (August-September), and 2013 (August-September). The corresponding households (women aged 15–49) samples were: 12,995 (15,029); 12,407 (13,983); 12,586 (13,633); 12,469 (13,594); and 14,804 (16,155), respectively. Complete birth history (CBH) modules were utilised as inputs into the mortality estimation. We assembled records on every child ever borne to female respondents, resulting in a combined dataset of 159,130 children ever borne under the age of five. Data quality was improved by deleting duplicates and removing observations that had unfeasible birth dates and death ages (e.g. children recorded to have died after the date of a survey interview).

Disparities in child mortality were examined across two dimensions: geography and socioeconomic position. In total three equity markers were utilised. Geographical-based disparities were measured using rural-urban location and grouping the country’s 17 regions into three island groups: Luzon, Visayas and Mindanao. Since the 1993 wave was not representative of all regions it was not used to compute the island estimates. Socioeconomic position was measured by wealth using the standard asset-based index provided in the DHS dataset [27]. To examine the data we divided an ordered distribution of the index into three parts, each containing a third of the population, that is, we created tertiles of the wealth index. The categories were denoted low, middle and high wealth. The robustness of the results to changes in classifications was tested by re-categorising wealth into quintiles.

### Mortality estimation and measures of disparities

Following the methods of Rajaratnam and colleagues, under-five and neonatal mortality, and associated 95% confidence intervals, at both national and sub-national levels, were estimated directly using pooled full birth history data structured into person-months [28]. Rates were estimated for two-year periods due to the relative rarity of child deaths using age-group mean survival probabilities and the associated survival rates. The estimation utilised sample weights and we accounted for the clustered sampling design using the Taylor-linearized-based variance estimator [29].

When assessing disparities the importance of the distinction between relative and absolute scales and their interpretation are well known [22,30,31]. In the scenario of falling overall rates of mortality, it is rather common to observe declines in the absolute differences in rates between two subpopulations over time while relative inequalities increase [20,32,33]. Accordingly, we calculate two measures on both scales. To capture absolute inequalities, rate difference (RD) and the slope index of inequality (SII) were computed. The rate ratio (RR) and the relative index of inequality (RII) were also estimated to gauge relative inequalities [30]. The RDs and RRs are straightforward differences and ratios between the mortality rates of each sub-population in reference to a base group, respectively. The measures were computed for each two-year period, providing a time series of inequality measures. For socioeconomic markers the highest ranked group was used as the base, while for the geography-related markers, the category over the sample period with the lowest average under-five mortality rate was the chosen referent. Instead of restricting measures to comparisons of extreme groups, the RIIs and SIIs use data on all sub-populations where the ordinal nature of the groups allows one to rank the population. In our case, this implies such indexes are only feasible for mortality rates stratified by wealth. These indexes were estimated via weighted linear regression [34]. This involves regressing mortality rates on the midpoint of the each sub-population rank on a scale of 0 to 1 determined by the cumulative population distribution of wealth. The main advantage of the RIIs and SIIs over the RDs and RRs is that these indexes account for any changes in the distribution of socioeconomic positions [35]. Further details on the measures of inequalities are provided in Text A in S1 File.

Similar to previous studies [6,36], confidence intervals on the mortality estimates and the RDs and RRs were constructed via simulation techniques [28,37]. In short, this involved generating 1,000 simulations of the survival probability for each time-period/age-group assuming a binomial distribution. The probability was set equal to the mean survival probability and sample size set to the number of person-months observed in the time-period/age-category. In each simulation, the rates of mortality and measures of disparities were then calculated for each time-period across each equity marker. The 2.5^th^ and 97.5^th^ percentiles were then used as the lower and upper confidence bounds. This standard simulation method captures both sampling and model uncertainty [28,37]. The 95% confidence intervals for the RIIs and SIIs were derived using standard methods outlined by Hayes and Berry [38].

We assessed changes over time by comparing mortality rates, rate differences and ratios, SIIs, and RIIs over the sample period to identify any patterns while accounting for the corresponding uncertainty in the measures. We also undertook tests of the statistical significance of a linear trend in these estimates [35]. When assessing the RRs and RIIs, we opted to use the natural logarithm of these measures in the trend regressions and report the exponentiated trend coefficients. Consequently, the reported figures can be interpreted as the average ratio change in RR or RII per period (e.g. a figure of 0.98 would imply the RR each year was expected to be 0.98 of last year). For RDs and SIIs, we report the trend coefficients directly; these coefficients can be interpreted as the average absolute change per period in the measures over the sample. Given the time series nature of the data and the possibility of correlation in the regression error terms, we estimated Newey-West standard errors (using one lag), which are robust to both heteroskedasticity and serial correlation [39]. All statistical analyses were conducted using *Stata* and *R*.

## Results

The mortality rates and estimates of absolute and relative inequality for each two-year period are presented in Tables A-D in S1 File. Despite some fluctuations between some two-year periods, consistent patterns over time are generally observed. These patterns are summarised below.

**Fig 1:**
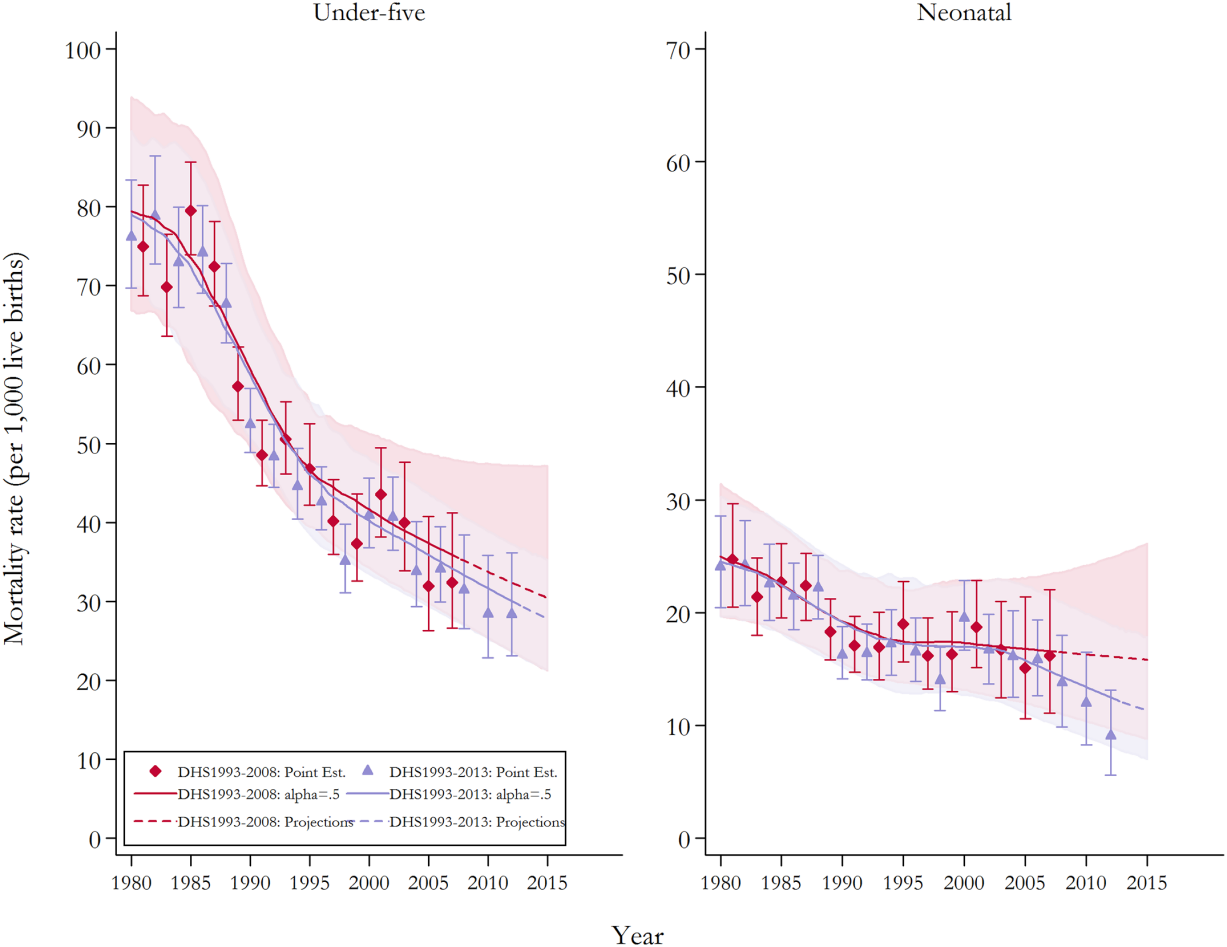
Under-five and neonatal mortality rates (per 1,000 live births) at the national level with and without the 2013 DHS wave. *Notes*: National estimates using pooled data are displayed with and without including DHS 2013. Loess regression using a smoothing parameter of 0.5 was applied to produce the continuous series.[53] The last set of parameter estimates for the Loess regression were utilised to project mortality rates toward 2015. The solid and semi-broken lines represent the continuous mortality estimates calculated from the two-year estimates, while the shaded area and area bars signify 95% confidence intervals. DHS, Demographic Health Survey; CI, confidence intervals

National estimates followed a general pattern of considerable declines in child mortality. Fig 1 presents the national estimates derived from pooling all DHS waves as well as excluding the most recent 2013 as a contrast. For U5MR, both sets of estimates provide a consistent pattern. Using the full set of data, under-five mortality has dropped from approximately 76 per 1,000 live deaths (95% CI 70 to 83) in 1980–81 to 53 (95% CI 49 to 57) in 1990–91 and 28 (95% CI 23 to 36) in 2012–13. The pattern of reduction in neonatal mortality differed. Following a reduction in NMR from 24 (95% CI 20 to 29) in 1980–81 to 16 (95% CI 14 to 19) in 1990–91, the rate of neonatal mortality remained relatively constant until 2006–07. This stagnation in neonatal mortality has been reported elsewhere [9]. However, our updated estimates using the most recent DHS wave show that progress in reducing neonatal morality has been achieved in recent years with NMR declining to 9 (95% CI 6 to 13) in 2012–13.

Despite the observed reductions the burden of mortality remains unequal. Table 1 presents the full range of inequality measures—both the absolute (i.e. RD and SII) and relative (i.e. RR and RII) measures—across wealth groups for selected years. We also report 95% confidence intervals as well as the trend coefficients and the corresponding *p*-values. As to be expected, low income households experience higher rates of under-five and neonatal mortality compared to richer households. The well-known patterns of reducing absolute but rising relative inequalities are found. However, the trends are statistically significant at conventional levels only with respect to absolute inequality in U5MR. For example, the RDs for under-five mortality have declined from 62.5 (95% CI 45.7 to 80.1–1.6 to 23.9) in 1980–81 to 31.5 (95% CI 5.5 to 41.1) in 2012–13. Note that the confidence intervals for the RIIs and SIIs estimates for the final time period are considerable. The final period estimates are based on data from only one survey and are calculated with the fewest observations. This might call into question their robustness and so we re-estimated the trend regressions excluding the final period. The trends of disparities remained unchanged except in the case of RRs for U5MR. However, as was the case previously, this trend is not statistically significant.

**Table 1:** Relative and absolute inequalities in under-five and neonatal mortality by wealth groups for selected years, with 95% confidence intervals and *p*-values for trend.

	Relative Inequalities				Absolute Inequalities			
	RR	95% CI	RII	95% CI	RD	95% CI	SII	95% CI
*Under-five mortality*								
1980–81	2.44	(1.85, 3.19)	3.93	(1.53, 6.32)	62.5	(45.7, 80.1)	93.03	(63.6, 122.46)
1990–91	3.12	(2.35, 3.94)	6.20	(3.69, 8.71)	51.6	(41.1, 61.3)	78.25	(68.47, 88.02)
2000–01	2.18	(1.57, 3.09)	3.94	(-16.83, 24.7)	30.7	(19.2, 41.7)	49.77	(-86.13, 185.68)
2010–11	2.22	(1.09, 4.23)	3.22	(-0.82, 7.26)	20.4	(2.7, 34)	30.81	(5.27, 56.35)
2012–13	3.04	(1.16, 3.81)	24.50	(-1638.84, 1687.84)	31.5	(5.5, 42.1)	55.59	(-221.34, 332.52)
Trend^1^ [*p*-value]	1.003	[0.631]	1.037	[0.247]	-2.502	[<0.000]	-3.470	[<0.000]
Trend^2^ [*p*-value]	0.999	[0.905]	1.004	[0.849]	-2.780	[<0.000]	-3.991	[<0.000]
*Neonatal mortality*								
1980–81	1.92	(1.28, 3.25)	2.92	(-6.29, 12.13)	15.6	(5.8, 25.7)	24.12	(-33.07, 81.31)
1990–91	1.42	(0.96, 2.1)	1.74	(-0.25, 3.73)	5.6	(-0.7, 11.4)	8.91	(-8.35, 26.16)
2000–01	2.09	(1.32, 3.99)	2.74	(-5.74, 11.22)	12.8	(5.2, 20.5)	18.45	(-28.12, 65.01)
2010–11	1.24	(0.56, 4.48)	1.35	(0.29, 2.41)	2.5	(-7.6, 12.6)	3.60	(-5.65, 12.84)
2012–13	2.17	(0.67, 7.64)	4.32	(-30.46, 39.09)	7.0	(-4.5, 14.7)	11.91	(-32.61, 56.42)
Trend^1^ [*p*-value]	1.009	[0.409]	1.018	[0.388]	-0.167	[0.407]	-0.252	[0.401]
Trend^2^ [*p*-value]	1.002	[0.845]	1.001	[0.948]	-0.207	[0.369]	-0.331	[0.332]

*Notes*: See S1 File for full results. CI, confidence interval; RR, rate ratio; RD, rate difference; RII, relative index of inequality; SII, slope index of inequality. The small number of observations and possible non-linear relationships implies that the trend estimates should be treated with caution. Additionally, since the bounds of the CI depend on the mean of mortality, comparisons over time must be treated cautiously.

^1.^ Trend regressions with all observations included.

^2.^ Trend regressions with the last 1 period (i.e. 2012–13) excluded from the sample. We report RDs and RRs for wealth as comparisons between the lowest and highest socioeconomic groups (i.e. lowest vs. highest wealth group). The results for the other group comparisons are available upon request.

**Fig 2:**
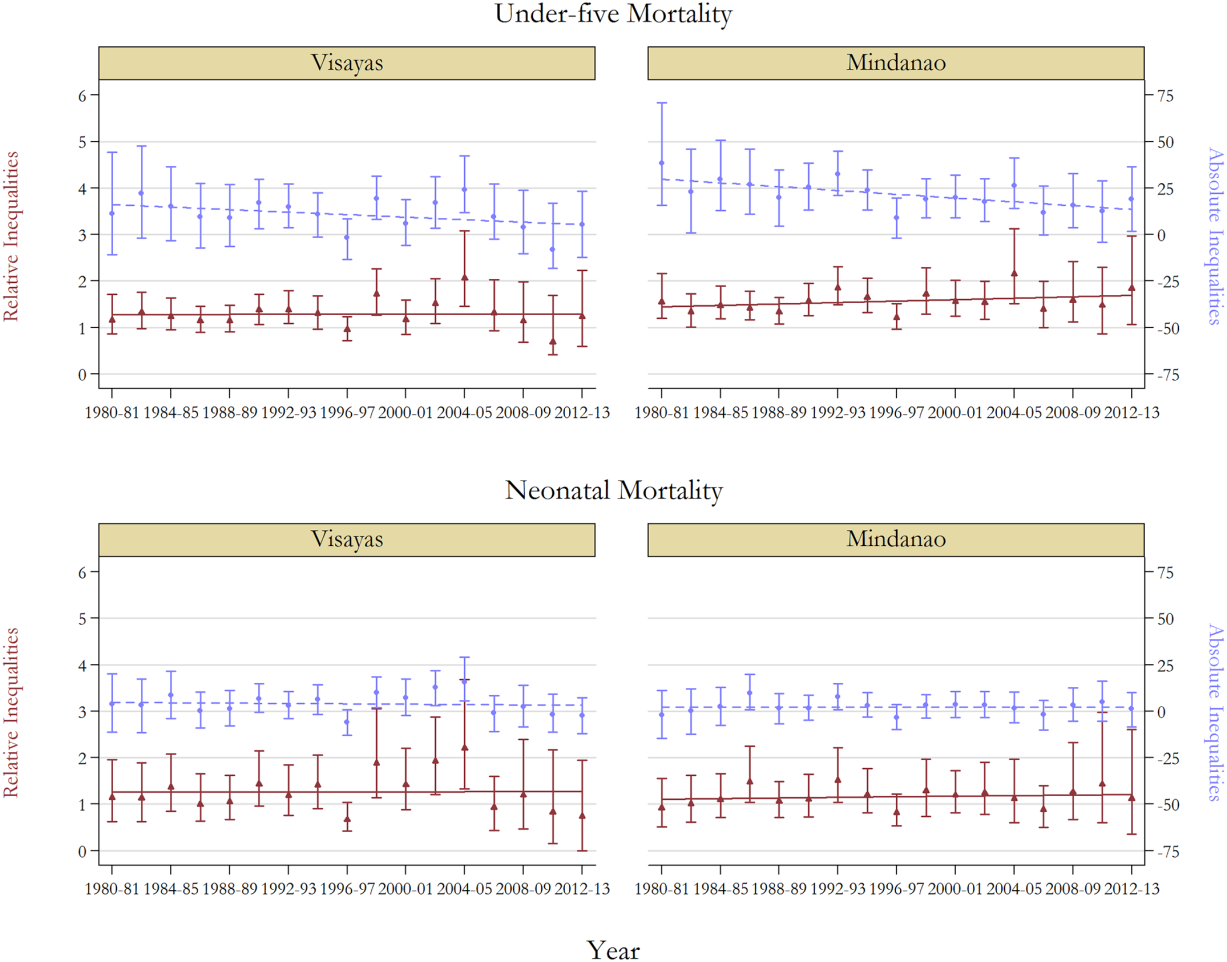
Trends in relative and absolute inequalities in mortality by island groups, with 95% confidence intervals, 1980–2013. *Notes*: See Table B in S1 File for full results. Base group is Luzon.

Table 2 presents measures of absolute (i.e. RD) and relative (i.e. RR) inequalities across rural-urban locations and islands. Again, measures are provided for NMR and U5MR and are reported with 95% confidence intervals for selected years as well as the linear trend coefficients of these measures over time with corresponding *p*-values. The results are a mixture, with few statistically significant results. For under-five mortality, absolute inequalities have shown significant downward trends across localities. For example, RDs by rural-urban location for under-five children decreased from 23.3 (95% CI 9.1 to 37.3) in 1980–81 to 10.6 (95% CI -1.6 to 26.4) in 2012–13. Relative inequalities appear to have remained stable, or perhaps increased. Divergent patterns are observed pertaining to the disparities in neonatal mortality. Absolute disparities were found to have increased over time. However, most trends were not statistically significant. On the other hand, rising relative inequalities were statistically significant at conventional levels across urban-rural locations. For example, RRs increased from 1.08 (95% CI 0.78 to 1.5) in 1980–89 to 2.71 (95% CI 1.3 to 9.8) in 2012–13. As shown in Fig 2, rising relative inequalities are also observed across island groups, although none are significant with respect to neonatal morality.

**Table 2:** Relative and absolute inequalities in under-five and neonatal mortality (per 1,000 live births) by rural-urban location and island divisions for selected years, with 95% confidence intervals and *p*-values for trend.

Equity Marker	Under-five Mortality				Neonatal Mortality			
	RR	95% CI	RD	95% CI	RR	95% CI	RD	95% CI
**Urban/Rural (base = Urban)**								
Rural								
1980–81	1.36	(1.13, 1.64)	23.3	(9.11, 37.32)	1.08	(0.78, 1.53)	1.7	(-5.91, 9.81)
1990–91	1.42	(1.2, 1.65)	17.9	(9.85, 25.94)	1.21	(0.91, 1.66)	3.0	(-1.65, 7.97)
2000–01	1.35	(1.07, 1.66)	12.1	(2.75, 20.26)	1.25	(0.89, 1.82)	4.3	(-2.08, 11.06)
2010–11	1.01	(0.63, 1.53)	0.2	(-13.94, 12.28)	1.23	(0.56, 2.75)	2.4	(-7.08, 10.29)
2012–13	1.46	(0.95, 2.56)	10.6	(-1.63, 26.36)	2.71	(1.27, 9.81)	8.2	(2.09, 16.28)
Trend^1^ [*p*-value]	0.998	[0.796]	-1.210	[<0.000]	1.028	[0.007]	0.146	[0.210]
Trend^2^ [*p*-value]	0.998	[0.809]	-1.267	[<0.000]	1.017	[0.054]	0.108	[0.468]
**Island Division (base = Luzon)**								
Visayas								
1980–81	1.16	(0.86, 1.71)	11.2	(-10.9, 44.2)	1.15	(0.62, 1.96)	3.7	(-11.23, 20.02)
1990–91	1.38	(1.06, 1.71)	17.0	(3.2, 29.6)	1.44	(0.96, 2.15)	6.6	(-0.68, 14.86)
2000–01	1.17	(0.85, 1.59)	5.8	(-5.9, 18.7)	1.42	(0.88, 2.2)	7.2	(-2.19, 17.38)
2010–11	0.68	(0.42, 1.69)	-8.4	(-18.2, 16.8)	0.83	(0.15, 2.17)	-1.9	(-11.33, 9.12)
2012–13	1.23	(0.59, 2.23)	5.1	(-12.4, 23.2)	0.74	(0, 1.95)	-2.4	(-12.04, 7.15)
Trend^1^ [*p*-value]	0.997	[0.801]	-0.676	[0.103]	0.994	[0.726]	-0.096	[0.722]
Trend^2^ [*p*-value]	0.996	[0.838]	-0.672	[0.200]	1.005	[0.756]	0.045	[0.881]
Mindanao								
1980–81	1.55	(1.2, 2.16)	38.3	(15.7, 70.9)	0.92	(0.51, 1.55)	-2.1	(-14.72, 11.09)
1990–91	1.57	(1.26, 1.95)	25.2	(13.1, 38.4)	1.10	(0.73, 1.65)	1.6	(-4.78, 8.51)
2000–01	1.56	(1.24, 2.01)	19.6	(9, 31.9)	1.19	(0.82, 1.72)	3.3	(-3.41, 10.6)
2010–11	1.48	(0.87, 2.3)	12.6	(-4.1, 28.7)	1.43	(0.6, 2.98)	4.7	(-5.4, 16.25)
2012–13	1.84	(1.06, 2.97)	18.8	(1.8, 36.5)	1.12	(0.35, 2.6)	1.1	(-8.53, 10.09)
Trend^1^ [*p*-value]	1.010	[0.044]	-1.018	[0.000]	1.006	[0.457]	0.009	[0.955]
Trend^2^ [*p*-value]	1.008	[0.176]	-1.149	[0.000]	1.008	[0.421]	0.041	[0.830]

*Notes*: See S1 File for full results. CI, confidence interval; RR, rate ratio; RD, rate difference. The small number of observations and possible non-linear relationships implies that the trend estimates should be treated with caution.

^1.^ Trend regressions with all observations included.

^2.^ Trend regressions with last 1 period (i.e. 2012–13) excluded from the sample.

## Discussion

The findings of our study, which utilises the most recently released data, show a national pattern contrary to previous studies [9,12]. In recent years, it is evident that the Philippines have achieved considerable success in reducing both U5MR and NMR at the national level. The laudable reduction of under-five mortality is mainly attributable to increased vaccination coverage (e.g. tetanus elimination and measles coverage) and programs addressing pneumonia and diarrhoea. While stagnation in the NMR has previously been observed, the updated estimates show reductions in neonatal deaths since 2006. This positive albeit surprising result might reflect the overall economic improvement experienced within the country in the previous decade as well as efforts to implement a nationwide universal health care campaign which was launched in 2010 [18,40–42].

Despite the positive downward trends in mortality levels as reported in Tables A-D in S1 File, inequalities between sub-populations remain. Unsurprisingly the burden of both under-five and neonatal mortality is disproportionately borne by the poor. While absolute inequalities with respect to wealth appear to have declined, relative inequalities have remained reasonably unchanged or possibly increased. This is a common pattern observed in similar contexts [20,32,33] specifically in emerging middle income countries with striking economic growth and simultaneous inequity increase [43]. In these settings where mortality rates are initially high, as shown in Tables A-D in S1 File for the Philippines, it is common to observe declining absolute inequalities, while relative inequalities remain stagnate. Notwithstanding, the success of the national insurance scheme (PhilHealth) in enrolling citizens from lower socioeconomic backgrounds, much remains to be done if the country is to tackle the existing levels of inequities. Recent studies suggest that increased insurance coverage has not automatically translated into lower wealth-related inequity in service utilization [43]. Barriers to care also remain [44] and it is likely that the poor continue to be disproportionally susceptible to risk factors associated with mortality, including low education and access to lower quality of care [9].

In 2015, the national health insurance program, Philhealth, launched a new and expanded Primary Care Benefits Package aimed to increase coverage of primary care. This package designed to address the most common health problems (such as pneumonia, diarrhea, hypertension and diabetes) targets the poorest in the country and will initially be rolled out to the bottom 40 percent of the population. However, without refocusing on and enhancing this equity focussed package to other emerging areas in child health, like malnutrition or prematurity, the package could have only a limited impact on equitable child mortality reduction.

Our study shows that despite improvements, across the urban/rural divide mortality is more severe in the rural sector. Inequality results across this equity marker are also mixed. Although a significant reduction in under-five absolute inequalities is reassuring, a significant increase in neonatal relative inequalities is a major concern. The persistent high levels of neonatal mortality in rural areas are likely explained by a number of factors. First, it is well known that the incidence, depth and severity of poverty are higher in rural than urban areas [45], and is likely having persistent effects on mortality outcomes. In addition to socioeconomic status, the mother’s age at time of her first birth, birth spacing, as well as reproductive ideals and norms in rural individuals and communities might be at play [46]. They are compounded by the limited health infrastructure—encompassing utilization of modern medicine and qualified personnel, access to clean water, sanitation, and environmental conditions—available in rural areas [46].

The Philippines features a highly decentralised health and governance system, with substantial authority, resources, and responsibilities being devolved from central level to sub-national units. The analysis should ideally focus at the municipal, city and provincial levels. However, our data is representative only at the regional level and reliable estimates are only available for the major island groups. For this marker we observed a significant downward trend in absolute inequalities for under-five deaths between the richer Luzon islands and the poorer Mindanao region and a marginally non statistically significant downward trend between Luzon and the central Visayas islands, [47]. All other inequalities remained relatively unchanged. While in principle decentralisation can be a powerful instrument in improving health service delivery, it can also pose significant risks and challenges. In addition to persistent poverty, continued inequalities potentially reflect on-going issues with low health sector infrastructure investments, the limited number and unequal distribution of health professionals and inaccessibility to health facilities in remote locations [48]. Efforts are needed to promote health leadership at subnational level, and enhance the practical implementation of child and newborn health care policies, particularly in disadvantaged locations. Standardising training curricula for mother and child health care providers around the country is also critical to improve the quality of care provided.

The regional disparities are being aggravated by severe typhoons which impact the Philippines every year, disrupting and destroying health facilities and newborn referral networks and eventually preventing the affected population from accessing quality health care services, sometimes even years after the typhoons’ impacts. Moreover, it is likely that armed conflict and civil unrest in the southern islands constrain further reductions in mortality levels. There is evidence that those countries where armed conflict takes place have a lower probability of achieving the MDGs [49]. Efforts need to be made to further advance the peace building process and ensure resilience of maternal, newborn and child health services against natural disasters to ensure that basic medical service provision is still accessible to the affected populations.

More generally, if the Philippines is to continue the path of improving neonatal health outcomes and inequities, two areas require attention. First, universal health coverage of skilled birth attendant should be given a priority. The most recent data suggests that only two thirds of all Filipino pregnant women deliver their newborn in the presence of a skilled birth attendant [18]. This would require a comprehensive health systems approach along the continuum of care which safeguards a woman’s adequate preconception care, while also guaranteeing the delivery of a healthy newborn [50]. Efforts must also be made to improve quality of care in order for women to be confident enough to access health care services [40,51]. Second, there is strong evidence that prevention of subsequent pregnancies in a woman’s lifetime is the best prevention for deaths in mothers and their newborns. While in December 2012 the Philippine Senate passed the Reproductive Health Law [52], efforts are currently being made to implement this law. Access to family planning services amongst the lowest quintile population could potentially prevent pregnancies and subsequent complications during child birth and comprehensively improve overall health outcomes. Additionally, implementation of the law would contribute to lowering the teenage pregnancy rate, which currently accounts for 13 percent of all pregnancies in the Philippines, and thus could have another positive impact on newborn mortality reduction.

A notable strength of this study is that the estimation of trends in both absolute and relative inequalities drawn from large, high-quality, nationally representative datasets with few missing observations. Two limitations, however, remain. First, for some sub-population estimates the associated uncertainty intervals are large, especially in the last two biennial periods [53]. Accordingly, in asserting the degree of inequality some caution is required, although the overall patterns of inequalities are likely to be accurate. Conversely, the statistical power of significance tests for linear trends is insufficient to detect small changes due to the limited number of data points. Yet, we have only emphasised consistent trends, also supported by other in-country evidence. Second, well-known measurement errors associated the potential influence of recall bias and with survey data may have affected the results. The pooling of data from multiple surveys should assist in addressing these potential biases and mitigate recall bias when surveys overlap.

This study provides a robust analysis of inequality trends in U5MR and NMR for the Philippines on both relative and absolute scales. It demonstrates that despite national progress made on reducing both mortality rates, disparities remain. Determined efforts are required to achieve universal coverage, account for the influence of decentralisation and armed conflict, and address issues along the continuum of care. Without continued surveillance and responsive policymaking disadvantaged sub-populations will continue to bear the burden of ill health and mortality.

## Supporting Information

S1 File Combined Supporting Information file containing: Text A, Measures of Relative and Absolute Inequality. Table A, Inequalities in under-five and neonatal mortality (per 1,000 live births) by wealth for all years, with 95% confidence intervals and p-values for trend. Table B, Inequalities in under-five and neonatal mortality (per 1,000 live births) by rural/urban location and regions for all years, with 95% confidence intervals and p-values for trend. Table C, Under-five mortality rates per 1,000 live births. Table D, Neonatal mortality rates per 1,000 live births.
(DOCX)
