# Correction: Overcoming Stagnation in the Levels and Distribution of Child Mortality: The Case of the Philippines

**DOI:** 10.1371/journal.pone.0141633

**Published:** 2015-10-21

**Authors:** Raoul Bermejo, Sonja Firth, Andrew Hodge, Eliana Jimenez-Soto, Willibald Zeck

The first affiliation for the fifth author is incorrect. Willibald Zeck is not affiliated with #2, but with #1: UNICEF Philippines, Makati City, Philippines.
