# Supplementary material for: Overcoming Stagnation in the Levels and Distribution of Child Mortality: The Case of the Philippines
Source: PLoS One. 2015 Oct 2;10(10):e0139458. doi: 10.1371/journal.pone.0139458 (PMC4592011; doi:10.1371/journal.pone.0139458)
Supplement: S1 File — Combined Supporting Information file containing: Text A, Measures of Relative and Absolute Inequality. Table A, Inequalities in under-five and neonatal mortality (per 1,000 live births) by wealth for all years, with 95% confidence intervals and p-values for trend. Table B, Inequalities in under-five and neonatal mortality (per 1,000 live births) by rural/urban location and regions for all years, with 95% confidence intervals and p-values for trend. Table C, Under-five mortality rates per 1,000 live births. Table D, Neonatal mortality rates per 1,000 live births. (DOCX) [file pone.0139458.s001.docx]

**File S1: Supporting Information**

**Text A: Measures of Relative and Absolute Inequality**

Rate Ratios (RR) and Rate Differences (RD)

Rate ratios and differences compare the outcomes between two groups on different scales. These groups are commonly defined by some socioeconomic characteristic (e.g. education); though, other indicators, such as geography (e.g. urban vs. rural), have also been utilised in previous studies. Often researchers are interested in the two extreme groups (e.g. lowest and highest wealth groups); however, any bi-group comparisons can be measured. For this study, these statistics are computed by taking simple ratios of or the difference between the mortality rates of the two groups.

Formally, let’s denote the two socioeconomic sub-populations as *j* = 1, 2, where the lowest socioeconomic group takes a value of 1 and the highest socioeconomic group a value of 2. The mortality rate for each group is denoted *MR_j_*. The rate ratio is computed as:

$$RR=\frac{MR_{1}}{MR_{2}}$$

And the rate difference is calculated as:

$$RD=MR_{1}-MR_{2}$$

The rate ratio provides a measure on inequality on a relative scale, while the rate difference provides an absolute measure.

Relative Index of Inequality (RII) and Slope Index of Inequality (SII)

The RII and SII are relative and absolute measures of inequality, respectively. Both take account of the population distribution categorised by socioeconomic status. Formally, let *j* = 1,…,*J* denote sub-populations ordered by socioeconomic status (e.g. by wealth). For each *j*, let *c_j_* be the fraction of the sample in group *j* or lower (with *c_0_* = 0 and *c_J_* = 1): that is, ordered by the socioeconomic marker, the cumulative relative position of the population. For each *j*, we define *ridit_j_* = (*c_j_* + *c_j-1_*)/2: that is, the midpoint of the relative rank. Finally, denote the mortality rate for each *j* as *MR_j_*. The measures are computed by first running a weighted least squares regression:

$$MR_{j}^{*}=\alpha+\beta ridit_{j}^{*}+\varepsilon_{j}$$

where the variables enter the regression transformed by weights, which in our study are the total person-months in each socioeconomic category. Using the estimated regression coefficients, the RII and SII are calculated as follows:

$$RII=\frac{\alpha}{\alpha+\beta}$$

and

$$SII=-\beta$$

**Table A : Inequalities in under-five and neonatal mortality (per 1,000 live births) by wealth for all years, with 95% confidence intervals and *p*-values for trend**

| **Equity Marker** |  | **Relative Inequalities** | | | |  | **Absolute Inequalities** | | | |
| --- | --- | --- | --- | --- | --- | --- | --- | --- | --- | --- |
|  |  | **RR** | **95% CI** | **RII** | **95% CI** |  | **RD** | **95% CI** | **SII** | **95% CI** |
| **Wealth** |  |  |  |  |  |  |  |  |  |  |
| *U5MR* |  |  |  |  |  |  |  |  |  |  |
| 1980-81 |  | 2.44 | (1.85; 3.19) | 3.93 | (1.53; 6.32) |  | 62.5 | (45.7; 80.1) | 93.03 | (63.6; 122.46) |
| 1982-83 |  | 2.19 | (1.7; 2.89) | 3.07 | (-5.25; 11.4) |  | 56.2 | (40.5; 72.1) | 82.02 | (-74.05; 238.09) |
| 1984-85 |  | 2.31 | (1.79; 2.89) | 3.20 | (-9.04; 15.43) |  | 53.9 | (39.4; 66.7) | 77.87 | (-121.31; 277.04) |
| 1986-87 |  | 2.61 | (2.06; 3.3) | 4.52 | (3.41; 5.63) |  | 64.1 | (50.9; 77.5) | 97.19 | (86.67; 107.71) |
| 1988-89 |  | 1.96 | (1.56; 2.36) | 3.12 | (-8.88; 15.13) |  | 44.5 | (31.1; 55.5) | 71.30 | (-117.01; 259.61) |
| 1990-91 |  | 3.12 | (2.35; 3.94) | 6.20 | (3.69; 8.71) |  | 51.6 | (41.1; 61.3) | 78.25 | (68.47; 88.02) |
| 1992-93 |  | 3.21 | (2.41; 4.28) | 6.47 | (5.14; 7.81) |  | 48.3 | (38.8; 57.8) | 73.15 | (68.7; 77.59) |
| 1994-95 |  | 3.39 | (2.47; 4.59) | 10.66 | (-106.4; 127.71) |  | 48.2 | (38.3; 58.7) | 76.57 | (-68.98; 222.12) |
| 1996-97 |  | 1.71 | (1.28; 2.29) | 2.92 | (-20.47; 26.3) |  | 23.8 | (11.7; 34.8) | 42.75 | (-215.03; 300.52) |
| 1998-99 |  | 2.67 | (1.8; 3.78) | 5.66 | (-26.03; 37.35) |  | 31.9 | (20.9; 41.3) | 50.67 | (-46.21; 147.55) |
| 2000-01 |  | 2.18 | (1.57; 3.09) | 3.94 | (-16.83; 24.7) |  | 30.7 | (19.2; 41.7) | 49.77 | (-86.13; 185.68) |
| 2002-03 |  | 2.78 | (1.94; 3.98) | 5.21 | (-0.18; 10.59) |  | 36.8 | (25.5; 47.3) | 56.79 | (34.78; 78.79) |
| 2004-05 |  | 3.06 | (1.7; 4.56) | 5.97 | (5.53; 6.4) |  | 32.5 | (18.7; 43.1) | 49.83 | (48.66; 51) |
| 2006-07 |  | 2.26 | (1.46; 3.55) | 4.39 | (-23.64; 32.42) |  | 26.7 | (13.9; 39.8) | 44.42 | (-84.71; 173.54) |
| 2008-09 |  | 2.22 | (1.23; 3.65) | 3.04 | (-4.88; 10.97) |  | 22.2 | (7.2; 33.9) | 32.71 | (-27.87; 93.29) |
| 2010-11 |  | 2.22 | (1.09; 4.23) | 3.22 | (-0.82; 7.26) |  | 20.4 | (2.7; 34) | 30.81 | (5.27; 56.35) |
| 2012-13 |  | 3.04 | (1.16; 3.81) | 24.50 | (-1638.84; 1687.84) |  | 31.5 | (5.5; 42.1) | 55.59 | (-221.34; 332.52) |
| Trend [*p*-value] |  | 1.003 | [0.631] | 1.037 | [0.247] |  | -2.502 | [<0.000] | -3.470 | [<0.000] |
|  |  |  |  |  |  |  |  |  |  |  |
| *NMR* |  |  |  |  |  |  |  |  |  |  |
| 1980-81 |  | 1.92 | (1.28; 3.25) | 2.92 | (-6.29; 12.13) |  | 15.6 | (5.8; 25.7) | 24.12 | (-33.07; 81.31) |
| 1982-83 |  | 1.30 | (0.82; 2.28) | 1.47 | (0.7; 2.24) |  | 6.3 | (-5.4; 16.6) | 9.24 | (-3.02; 21.5) |
| 1984-85 |  | 1.45 | (0.99; 2.24) | 1.56 | (-5.36; 8.48) |  | 7.6 | (-0.3; 15) | 9.90 | (-85.31; 105.12) |
| 1986-87 |  | 1.22 | (0.84; 1.78) | 1.48 | (-4.33; 7.29) |  | 4.5 | (-4.2; 11.9) | 8.36 | (-72.96; 89.67) |
| 1988-89 |  | 1.02 | (0.72; 1.41) | 1.16 | (-5.28; 7.59) |  | 0.4 | (-8.4; 7.9) | 3.26 | (-119.79; 126.3) |
| 1990-91 |  | 1.42 | (0.96; 2.1) | 1.74 | (-0.25; 3.73) |  | 5.6 | (-0.7; 11.4) | 8.91 | (-8.35; 26.16) |
| 1992-93 |  | 1.75 | (1.15; 2.73) | 2.00 | (-5.87; 9.86) |  | 8.2 | (2.2; 13.3) | 11.03 | (-46.1; 68.16) |
| 1994-95 |  | 2.19 | (1.41; 3.52) | 3.20 | (1.48; 4.92) |  | 12.3 | (5.7; 18.8) | 18.43 | (11.83; 25.04) |
| 1996-97 |  | 1.00 | (0.65; 1.62) | 1.37 | (-16.18; 18.92) |  | 0.0 | (-9.5; 8.2) | 5.20 | (-201.69; 212.09) |
| 1998-99 |  | 1.54 | (0.93; 2.74) | 2.39 | (-15.87; 20.65) |  | 6.4 | (-1.2; 13) | 11.68 | (-76.55; 99.92) |
| 2000-01 |  | 2.09 | (1.32; 3.99) | 2.74 | (-5.74; 11.22) |  | 12.8 | (5.2; 20.5) | 18.45 | (-28.12; 65.01) |
| 2002-03 |  | 1.59 | (0.97; 2.72) | 1.91 | (-1.28; 5.11) |  | 7.3 | (-0.5; 14.1) | 10.59 | (-14.54; 35.71) |
| 2004-05 |  | 1.92 | (1; 4.03) | 2.68 | (2.63; 2.73) |  | 9.8 | (0.1; 18.4) | 14.99 | (14.74; 15.24) |
| 2006-07 |  | 1.30 | (0.73; 2.88) | 1.47 | (0.82; 2.12) |  | 4.1 | (-5.5; 13.8) | 6.07 | (-0.71; 12.85) |
| 2008-09 |  | 1.44 | (0.69; 3.15) | 1.73 | (1.6; 1.87) |  | 4.9 | (-5.6; 12.9) | 7.53 | (6.53; 8.53) |
| 2010-11 |  | 1.24 | (0.56; 4.48) | 1.35 | (0.29; 2.41) |  | 2.5 | (-7.6; 12.6) | 3.60 | (-5.65; 12.84) |
| 2012-13 |  | 2.17 | (0.67; 7.64) | 4.32 | (-30.46; 39.09) |  | 7.0 | (-4.5; 14.7) | 11.91 | (-32.61; 56.42) |
| Trend [*p*-value] |  | 1.009 | [0.409] | 1.018 | [0.388] |  | -0.167 | [0.407] | -0.252 | [0.401] |

*Notes*: U5MR, under-five mortality rate; NMR, neonatal mortality rate; CI, confidence interval; RR, rate ratio; RD, rate difference; RII, relative index of inequality; SII, slope index of inequality. The small number of observations and possible non-linear relationships implies that the trend estimates should be treated with caution..

**Table B : Inequalities in under-five and neonatal mortality (per 1,000 live births) by rural/urban location and regions for all years, with 95% confidence intervals and *p*-values for trend**

| **Equity Marker** |  | **U5MR** | | | |  | **NMR** | | | |
| --- | --- | --- | --- | --- | --- | --- | --- | --- | --- | --- |
|  |  | **RR** | **95% CI** | **RD** | **95% CI** |  | **RR** | **95% CI** | **RD** | **95% CI** |
| **Urban/Rural (base = Urban)** | | | |  |  |  |  |  |  |  |
| Rural |  |  |  |  |  |  |  |  |  |  |
| 1980-81 |  | 1.36 | (1.13; 1.64) | 23.3 | (9.11; 37.32) |  | 1.08 | (0.78; 1.53) | 1.7 | (-5.91; 9.81) |
| 1982-83 |  | 1.47 | (1.25; 1.75) | 29.7 | (17.39; 43.14) |  | 1.19 | (0.86; 1.62) | 4.1 | (-3.79; 11.18) |
| 1984-85 |  | 1.38 | (1.18; 1.63) | 23.1 | (12.05; 33.85) |  | 1.39 | (1.05; 1.85) | 7.3 | (1.2; 12.82) |
| 1986-87 |  | 1.46 | (1.26; 1.72) | 27.5 | (17.35; 39.06) |  | 1.36 | (1.03; 1.89) | 6.5 | (0.72; 12.58) |
| 1988-89 |  | 1.45 | (1.23; 1.68) | 24.6 | (14.27; 33.87) |  | 1.24 | (0.93; 1.61) | 4.7 | (-1.65; 10.01) |
| 1990-91 |  | 1.42 | (1.2; 1.65) | 17.9 | (9.85; 25.94) |  | 1.21 | (0.91; 1.66) | 3.0 | (-1.65; 7.97) |
| 1992-93 |  | 1.70 | (1.42; 2.06) | 24.7 | (16.93; 32.88) |  | 1.59 | (1.15; 2.22) | 7.3 | (2.49; 12.09) |
| 1994-95 |  | 1.68 | (1.39; 2.07) | 22.2 | (14.5; 30.4) |  | 1.68 | (1.21; 2.42) | 8.6 | (3.14; 14.11) |
| 1996-97 |  | 1.19 | (0.95; 1.46) | 7.3 | (-1.99; 15.86) |  | 1.18 | (0.83; 1.71) | 2.8 | (-3.25; 8.47) |
| 1998-99 |  | 1.93 | (1.45; 2.51) | 21.8 | (12.82; 29.89) |  | 1.10 | (0.72; 1.66) | 1.4 | (-4.92; 7.1) |
| 2000-01 |  | 1.35 | (1.07; 1.66) | 12.1 | (2.75; 20.26) |  | 1.25 | (0.89; 1.82) | 4.3 | (-2.08; 11.06) |
| 2002-03 |  | 1.51 | (1.19; 1.91) | 16.3 | (7.1; 25.65) |  | 1.44 | (1; 2.1) | 6.0 | (-0.01; 11.93) |
| 2004-05 |  | 1.58 | (1.19; 2.29) | 15.1 | (5.72; 26.45) |  | 2.07 | (1.26; 3.63) | 11.0 | (3.65; 18.63) |
| 2006-07 |  | 1.53 | (1.14; 2.14) | 14.1 | (4.74; 25.12) |  | 1.48 | (0.96; 2.46) | 6.1 | (-0.62; 13.84) |
| 2008-09 |  | 1.62 | (1.09; 2.29) | 14.6 | (2.92; 26.3) |  | 1.76 | (0.94; 3.41) | 7.5 | (-0.84; 15.08) |
| 2010-11 |  | 1.01 | (0.63; 1.53) | 0.2 | (-13.94; 12.28) |  | 1.23 | (0.56; 2.75) | 2.4 | (-7.08; 10.29) |
| 2012-13 |  | 1.46 | (0.95; 2.56) | 10.6 | (-1.63; 26.36) |  | 2.71 | (1.27; 9.81) | 8.2 | (2.09; 16.28) |
| Trend [*p*-value] |  | 0.998 | [0.796] | -1.210 | [<0.000] |  | 1.028 | [0.007] | 0.146 | [0.210] |
|  |  |  |  |  |  |  |  |  |  |  |
| **Island Division (base = Luzon)** | | | |  |  |  |  |  |  |  |
| Visayas |  |  |  |  |  |  |  |  |  |  |
| 1980-81 |  | 1.16 | (0.86; 1.71) | 11.2 | (-10.87; 44.16) |  | 1.15 | (0.62; 1.96) | 3.7 | (-11.23; 20.02) |
| 1982-83 |  | 1.32 | (0.98; 1.76) | 21.9 | (-1.9; 47.74) |  | 1.14 | (0.62; 1.88) | 3.3 | (-11.46; 17.22) |
| 1984-85 |  | 1.24 | (0.95; 1.63) | 14.9 | (-3.29; 36.35) |  | 1.36 | (0.85; 2.07) | 8.5 | (-3.98; 21.46) |
| 1986-87 |  | 1.14 | (0.9; 1.45) | 9.3 | (-7.26; 27.49) |  | 1.00 | (0.63; 1.66) | 0.0 | (-8.93; 10.23) |
| 1988-89 |  | 1.15 | (0.9; 1.48) | 8.7 | (-6.53; 26.86) |  | 1.06 | (0.67; 1.62) | 1.2 | (-7.87; 11.24) |
| 1990-91 |  | 1.38 | (1.06; 1.71) | 17.0 | (3.18; 29.61) |  | 1.44 | (0.96; 2.15) | 6.6 | (-0.68; 14.86) |
| 1992-93 |  | 1.38 | (1.09; 1.8) | 14.6 | (3.67; 27.26) |  | 1.19 | (0.76; 1.85) | 2.8 | (-3.91; 10.45) |
| 1994-95 |  | 1.30 | (0.97; 1.68) | 10.9 | (-1.31; 22.3) |  | 1.41 | (0.91; 2.06) | 6.3 | (-1.58; 14.26) |
| 1996-97 |  | 0.95 | (0.72; 1.23) | -1.9 | (-13.46; 8.57) |  | 0.67 | (0.42; 1.04) | -6.2 | (-13.01; 0.75) |
| 1998-99 |  | 1.72 | (1.27; 2.26) | 19.1 | (8.05; 31.31) |  | 1.88 | (1.14; 3.06) | 9.9 | (1.81; 18.54) |
| 2000-01 |  | 1.17 | (0.85; 1.59) | 5.8 | (-5.94; 18.68) |  | 1.42 | (0.88; 2.2) | 7.2 | (-2.19; 17.38) |
| 2002-03 |  | 1.51 | (1.09; 2.04) | 17.0 | (3.3; 31.02) |  | 1.93 | (1.2; 2.87) | 12.6 | (3.12; 21.66) |
| 2004-05 |  | 2.06 | (1.45; 3.08) | 23.9 | (11.85; 42.2) |  | 2.21 | (1.33; 3.69) | 15.5 | (5.49; 29) |
| 2006-07 |  | 1.32 | (0.93; 2.02) | 9.3 | (-2.44; 27.31) |  | 0.93 | (0.43; 1.6) | -1.2 | (-11.01; 8.37) |
| 2008-09 |  | 1.14 | (0.68; 1.99) | 3.7 | (-10.23; 23.91) |  | 1.19 | (0.47; 2.4) | 2.4 | (-8.56; 13.95) |
| 2010-11 |  | 0.68 | (0.42; 1.69) | -8.4 | (-18.24; 16.84) |  | 0.83 | (0.15; 2.17) | -1.9 | (-11.33; 9.12) |
| 2012-13 |  | 1.23 | (0.59; 2.23) | 5.1 | (-12.39; 23.22) |  | 0.74 | (0; 1.95) | -2.4 | (-12.04; 7.15) |
| Trend [*p*-value] |  | 0.997 | [0.801] | -0.676 | [0.103] |  | 0.994 | [0.726] | -0.096 | [0.722] |
|  |  |  |  |  |  |  |  |  |  |  |
| Mindanao |  |  |  |  |  |  |  |  |  |  |
| 1980-81 |  | 1.55 | (1.2; 2.16) | 38.3 | (15.72; 70.86) |  | 0.92 | (0.51; 1.55) | -2.1 | (-14.72; 11.09) |
| 1982-83 |  | 1.33 | (1.01; 1.73) | 22.7 | (0.92; 46.03) |  | 1.01 | (0.61; 1.62) | 0.2 | (-12.49; 12) |
| 1984-85 |  | 1.47 | (1.18; 1.89) | 29.5 | (12.85; 50.71) |  | 1.09 | (0.71; 1.65) | 2.2 | (-7.65; 12.73) |
| 1986-87 |  | 1.41 | (1.16; 1.77) | 26.8 | (10.93; 45.82) |  | 1.48 | (1.04; 2.25) | 9.5 | (0.83; 19.92) |
| 1988-89 |  | 1.33 | (1.07; 1.64) | 19.9 | (4.61; 34.68) |  | 1.06 | (0.72; 1.49) | 1.3 | (-6.9; 9.4) |
| 1990-91 |  | 1.57 | (1.26; 1.95) | 25.2 | (13.09; 38.37) |  | 1.10 | (0.73; 1.65) | 1.6 | (-4.78; 8.51) |
| 1992-93 |  | 1.85 | (1.49; 2.31) | 32.3 | (20.93; 44.82) |  | 1.52 | (1.04; 2.21) | 7.5 | (0.81; 14.74) |
| 1994-95 |  | 1.65 | (1.32; 2.06) | 23.7 | (13.15; 34.67) |  | 1.19 | (0.81; 1.77) | 3.0 | (-3.2; 9.96) |
| 1996-97 |  | 1.22 | (0.96; 1.51) | 8.9 | (-2.05; 19.58) |  | 0.82 | (0.53; 1.22) | -3.4 | (-9.92; 3.57) |
| 1998-99 |  | 1.71 | (1.29; 2.28) | 18.9 | (9; 29.94) |  | 1.28 | (0.73; 1.96) | 3.2 | (-3.66; 8.95) |
| 2000-01 |  | 1.56 | (1.24; 2.01) | 19.6 | (9.03; 31.9) |  | 1.19 | (0.82; 1.72) | 3.3 | (-3.41; 10.6) |
| 2002-03 |  | 1.53 | (1.18; 2) | 17.6 | (6.96; 29.89) |  | 1.24 | (0.78; 1.9) | 3.3 | (-3.43; 10.52) |
| 2004-05 |  | 2.15 | (1.51; 3.12) | 26.1 | (13.9; 41.09) |  | 1.12 | (0.6; 1.96) | 1.6 | (-6.19; 10.24) |
| 2006-07 |  | 1.39 | (1; 2) | 11.6 | (-0.16; 26.09) |  | 0.88 | (0.5; 1.4) | -2.0 | (-10.24; 5.97) |
| 2008-09 |  | 1.58 | (1.12; 2.42) | 15.5 | (3.55; 32.91) |  | 1.26 | (0.67; 2.32) | 3.3 | (-5.35; 12.55) |
| 2010-11 |  | 1.48 | (0.87; 2.3) | 12.6 | (-4.15; 28.74) |  | 1.43 | (0.6; 2.98) | 4.7 | (-5.4; 16.25) |
| 2012-13 |  | 1.84 | (1.06; 2.97) | 18.8 | (1.77; 36.53) |  | 1.12 | (0.35; 2.6) | 1.1 | (-8.53; 10.09) |
| Trend [*p*-value] |  | 1.010 | [0.044] | -1.018 | [<0.000] |  | 1.006 | [0.457] | 0.009 | [0.955] |

*Notes*: U5MR, under-five mortality rate; NMR, neonatal mortality rate; CI, confidence interval; RR, rate ratio; RD, rate difference. The small number of observations and possible non-linear relationships implies that the trend estimates should be treated with caution.

**Table C: Under-five mortality rates per 1,000 live births**

| **Equity Marker** | **U5MR** | **95% CI** | **U5MR** | **95% CI** | **U5MR** | **95% CI** |
| --- | --- | --- | --- | --- | --- | --- |
| **National** | National |  |  |  |  |  |
| 1980-81 | 76.2 | (69.7; 83.4) |  |  |  |  |
| 1982-83 | 78.8 | (72.8; 86.4) |  |  |  |  |
| 1984-85 | 72.9 | (67.2; 79.9) |  |  |  |  |
| 1986-87 | 74.2 | (69; 80.1) |  |  |  |  |
| 1988-89 | 67.6 | (62.7; 72.8) |  |  |  |  |
| 1990-91 | 52.4 | (48.9; 56.9) |  |  |  |  |
| 1992-93 | 48.3 | (44.5; 52.4) |  |  |  |  |
| 1994-95 | 44.5 | (40.4; 49.4) |  |  |  |  |
| 1996-97 | 42.6 | (39.1; 47.1) |  |  |  |  |
| 1998-99 | 35.1 | (31.1; 39.8) |  |  |  |  |
| 2000-01 | 40.9 | (36.8; 45.6) |  |  |  |  |
| 2002-03 | 40.7 | (36.5; 45.8) |  |  |  |  |
| 2004-05 | 33.8 | (29.3; 40.1) |  |  |  |  |
| 2006-07 | 34.1 | (29.9; 39.5) |  |  |  |  |
| 2008-09 | 31.4 | (26.6; 38.4) |  |  |  |  |
| 2010-11 | 28.4 | (22.9; 35.9) |  |  |  |  |
| 2012-13 | 28.3 | (23.1; 36.1) |  |  |  |  |
| Trend [*p*-value] | -3.38 | [<0.000] |  |  |  |  |
|  |  |  |  |  |  |  |
| **Urban/Rural** | Urban |  | Rural |  |  |  |
| 1980-81 | 63.9 | (54.9; 74.1) | 87.2 | (77.9; 98.4) |  |  |
| 1982-83 | 63.3 | (55.3; 73.2) | 92.9 | (84.7; 104.1) |  |  |
| 1984-85 | 60.6 | (53.4; 69.5) | 83.7 | (76.2; 92.7) |  |  |
| 1986-87 | 59.4 | (52; 67.9) | 86.9 | (79.4; 95.5) |  |  |
| 1988-89 | 54.4 | (48.5; 62.1) | 79.0 | (72.5; 86.9) |  |  |
| 1990-91 | 42.9 | (37.7; 49.7) | 60.8 | (55.7; 67.3) |  |  |
| 1992-93 | 35.1 | (30.2; 41.4) | 59.8 | (54.4; 66.5) |  |  |
| 1994-95 | 32.5 | (27.4; 38.7) | 54.7 | (49.2; 61.8) |  |  |
| 1996-97 | 38.7 | (32.8; 46.1) | 46.0 | (40.5; 52.6) |  |  |
| 1998-99 | 23.5 | (19.1; 30) | 45.4 | (39.9; 52.5) |  |  |
| 2000-01 | 34.6 | (30; 42.4) | 46.7 | (41; 53.9) |  |  |
| 2002-03 | 32.2 | (26.9; 39.4) | 48.5 | (43; 56.4) |  |  |
| 2004-05 | 25.8 | (19.4; 32.5) | 40.9 | (34.4; 50) |  |  |
| 2006-07 | 26.7 | (20.5; 34.7) | 40.9 | (34.7; 49.6) |  |  |
| 2008-09 | 23.7 | (18.5; 34.3) | 38.3 | (31.7; 49.6) |  |  |
| 2010-11 | 28.3 | (21.7; 41.7) | 28.5 | (22; 39.2) |  |  |
| 2012-13 | 22.8 | (14.9; 32.9) | 33.4 | (26.5; 46.6) |  |  |
| Trend [*p*-value] | -2.73 | [<0.000] | -3.94 | [<0.000] |  |  |
|  |  |  |  |  |  |  |
| **Wealth** | Low |  | Middle |  | High |  |
| 1980-81 | 106.0 | (95.1; 120.7) | 72.9 | (63.3; 87.5) | 43.5 | (34.2; 55.2) |
| 1982-83 | 103.4 | (93.2; 115.9) | 79.5 | (69.7; 91.6) | 47.1 | (37; 60.2) |
| 1984-85 | 95.0 | (85.8; 106) | 74.0 | (65; 84.7) | 41.1 | (33.9; 51.7) |
| 1986-87 | 103.9 | (95; 115.1) | 66.9 | (59.2; 77) | 39.8 | (32.6; 50.1) |
| 1988-89 | 90.9 | (82.4; 101.2) | 56.4 | (49.5; 65.8) | 46.4 | (39; 57.3) |
| 1990-91 | 76.0 | (68.7; 84.8) | 45.6 | (39.8; 53.9) | 24.4 | (19.9; 31.9) |
| 1992-93 | 70.2 | (63.9; 77.8) | 41.9 | (36.6; 49.6) | 21.9 | (17.1; 28.6) |
| 1994-95 | 68.4 | (61.5; 77.4) | 33.3 | (27.5; 40.4) | 20.2 | (15.7; 27.4) |
| 1996-97 | 57.3 | (50.9; 65.4) | 30.9 | (25.2; 38.1) | 33.5 | (25.4; 44.3) |
| 1998-99 | 51.0 | (44.6; 59.2) | 27.9 | (21.6; 35.9) | 19.1 | (13.9; 27.7) |
| 2000-01 | 56.7 | (50.6; 65.8) | 32.4 | (26; 40.7) | 26.0 | (19.3; 35.6) |
| 2002-03 | 57.5 | (50.2; 67.3) | 34.9 | (28.7; 43.8) | 20.7 | (15.4; 28.6) |
| 2004-05 | 48.2 | (40.4; 59.3) | 29.1 | (21.8; 38.9) | 15.7 | (10.7; 28.3) |
| 2006-07 | 48.0 | (41.3; 58.9) | 25.8 | (19.7; 35.6) | 21.2 | (14.4; 32.3) |
| 2008-09 | 40.4 | (33.6; 51.6) | 30.0 | (21.3; 42.9) | 18.2 | (12; 32.3) |
| 2010-11 | 37.0 | (29.7; 50.5) | 26.0 | (19; 42.6) | 16.6 | (9.9; 33.4) |
| 2012-13 | 46.9 | (37; 62.7) | 14.7 | (9.7; 28.6) | 15.4 | (12.9; 39.7) |
| Trend [*p*-value] | -4.45 | [<0.000] | -3.61 | [<0.000] | -1.95 | [<0.000] |
|  |  |  |  |  |  |  |
| **Island** | Luzon |  | Visayas |  | Mindanao |  |
| 1980-81 | 69.2 | (56.3; 86.2) | 80.4 | (64.4; 110.8) | 107.5 | (91; 136.8) |
| 1982-83 | 68.1 | (57.5; 85.1) | 90.1 | (72.8; 114.4) | 90.8 | (76.7; 112.4) |
| 1984-85 | 62.8 | (53.6; 74.5) | 77.7 | (64.3; 97.5) | 92.4 | (79.8; 111.3) |
| 1986-87 | 64.6 | (55.3; 75.2) | 73.8 | (61.3; 90.3) | 91.3 | (78.5; 109) |
| 1988-89 | 59.7 | (52.3; 69.5) | 68.4 | (56.9; 85.4) | 79.5 | (68.4; 93.9) |
| 1990-91 | 44.2 | (38.6; 53.2) | 61.2 | (51.1; 73.3) | 69.5 | (60.6; 82.3) |
| 1992-93 | 38.1 | (32.4; 44.5) | 52.7 | (43.8; 64.9) | 70.4 | (61.3; 82.4) |
| 1994-95 | 36.3 | (31.3; 42.7) | 47.2 | (37.8; 57.8) | 60.0 | (51.6; 70.4) |
| 1996-97 | 40.7 | (35.4; 48) | 38.8 | (31.3; 48.3) | 49.6 | (42.5; 59.3) |
| 1998-99 | 26.5 | (22.2; 32.5) | 45.6 | (36.4; 57.4) | 45.4 | (37.6; 55.8) |
| 2000-01 | 34.8 | (29.5; 41.2) | 40.5 | (31.2; 52.6) | 54.4 | (46.5; 64.9) |
| 2002-03 | 33.0 | (27.7; 39.8) | 50.0 | (38.3; 63.2) | 50.6 | (42.1; 62) |
| 2004-05 | 22.6 | (17.7; 29.5) | 46.5 | (35.9; 64.2) | 48.6 | (38.2; 62.8) |
| 2006-07 | 29.4 | (23.8; 37.3) | 38.7 | (29.1; 57.1) | 41.0 | (33.2; 55.4) |
| 2008-09 | 26.7 | (20.2; 35.5) | 30.4 | (21.1; 49.7) | 42.2 | (33.9; 58.6) |
| 2010-11 | 26.4 | (19.5; 35.3) | 18.1 | (12.3; 42) | 39.0 | (26.1; 54.5) |
| 2012-13 | 22.4 | (16.6; 34.2) | 27.5 | (15.6; 45.4) | 41.2 | (30.1; 58.6) |
| Trend [*p*-value] | -3.08 | [<0.000] | -3.76 | [<0.000] | -4.10 | [<0.000] |

*Notes*: U5MR, under-five mortality rate; CI, confidence interval; Quin., Quintile. The small number of observations and possible non-linear relationships implies that the trend estimates should be treated with caution.

**Table D: Neonatal mortality rates per 1,000 live births**

| **Equity Marker** | **NMR** | **95% CI** | **NMR** | **95% CI** | **NMR** | **95% CI** |
| --- | --- | --- | --- | --- | --- | --- |
| **National** | National |  |  |  |  |  |
| 1980-81 | 24.1 | (20.4; 28.6) |  |  |  |  |
| 1982-83 | 24.2 | (20.6; 28.2) |  |  |  |  |
| 1984-85 | 22.6 | (19.3; 26.1) |  |  |  |  |
| 1986-87 | 21.5 | (18.5; 24.4) |  |  |  |  |
| 1988-89 | 22.2 | (19.4; 25.1) |  |  |  |  |
| 1990-91 | 16.2 | (14.1; 18.8) |  |  |  |  |
| 1992-93 | 16.4 | (14; 19) |  |  |  |  |
| 1994-95 | 17.3 | (14.5; 20.3) |  |  |  |  |
| 1996-97 | 16.5 | (13.9; 19.5) |  |  |  |  |
| 1998-99 | 14.0 | (11.3; 16.9) |  |  |  |  |
| 2000-01 | 19.5 | (16.7; 22.8) |  |  |  |  |
| 2002-03 | 16.7 | (13.7; 19.9) |  |  |  |  |
| 2004-05 | 16.1 | (12.5; 20.2) |  |  |  |  |
| 2006-07 | 15.8 | (12.6; 19.3) |  |  |  |  |
| 2008-09 | 13.8 | (9.9; 18) |  |  |  |  |
| 2010-11 | 12.0 | (8.3; 16.5) |  |  |  |  |
| 2012-13 | 9.1 | (5.6; 13.1) |  |  |  |  |
| Trend [*p*-value] | -0.75 | [<0.000] |  |  |  |  |
|  |  |  |  |  |  |  |
| **Urban/Rural** | Urban |  | Rural |  |  |  |
| 1980-81 | 24.9 | (19.7; 30.5) | 23.2 | (17.6; 29.1) |  |  |
| 1982-83 | 26.2 | (21.1; 31.7) | 22.0 | (16.4; 28.5) |  |  |
| 1984-85 | 25.9 | (21.5; 30.4) | 18.7 | (14.6; 23.4) |  |  |
| 1986-87 | 24.5 | (20.7; 28.8) | 18.0 | (13.5; 22.6) |  |  |
| 1988-89 | 24.4 | (20.3; 28.3) | 19.7 | (16; 24.8) |  |  |
| 1990-91 | 17.7 | (14.7; 21.1) | 14.7 | (11.4; 18.4) |  |  |
| 1992-93 | 19.8 | (16.5; 23.4) | 12.4 | (9.2; 15.9) |  |  |
| 1994-95 | 21.2 | (17.3; 25.7) | 12.6 | (9.1; 16.3) |  |  |
| 1996-97 | 17.8 | (14.4; 21.6) | 15.1 | (11; 20.1) |  |  |
| 1998-99 | 14.7 | (11.2; 18.7) | 13.3 | (9.3; 18) |  |  |
| 2000-01 | 21.6 | (17.2; 26.7) | 17.3 | (13.1; 22.5) |  |  |
| 2002-03 | 19.6 | (15.3; 24) | 13.6 | (9.9; 17.7) |  |  |
| 2004-05 | 21.3 | (15.9; 27.4) | 10.3 | (6.1; 14.5) |  |  |
| 2006-07 | 18.7 | (14.1; 24.7) | 12.6 | (8.1; 17.9) |  |  |
| 2008-09 | 17.3 | (12.2; 24) | 9.8 | (5.5; 15.9) |  |  |
| 2010-11 | 13.1 | (7.7; 19.3) | 10.7 | (6; 18.2) |  |  |
| 2012-13 | 13.0 | (7.3; 20.1) | 4.8 | (1.7; 8.7) |  |  |
| Trend [*p*-value] | -0.83 | [<0.000] | -0.68 | [<0.000] |  |  |
|  |  |  |  |  |  |  |
| **Wealth** | Low |  | Middle |  | High |  |
| 1980-81 | 32.5 | (25.1; 40.5) | 21.3 | (15.4; 28.9) | 16.9 | (10.9; 23.5) |
| 1982-83 | 26.9 | (20.8; 33.6) | 24.0 | (18.7; 30) | 20.6 | (11.7; 31.1) |
| 1984-85 | 24.6 | (19.7; 29.9) | 24.7 | (18.7; 30.8) | 16.9 | (11.5; 22.8) |
| 1986-87 | 24.8 | (20.7; 29.6) | 18.4 | (13.5; 23.4) | 20.3 | (14.3; 27.7) |
| 1988-89 | 24.3 | (19.8; 29) | 18.3 | (14.2; 22.7) | 23.9 | (17.8; 31) |
| 1990-91 | 19.1 | (15.5; 23) | 15.0 | (11.3; 18.8) | 13.4 | (9.6; 18.4) |
| 1992-93 | 19.1 | (15.4; 22.9) | 17.1 | (13.4; 22.2) | 11.0 | (7.2; 15.7) |
| 1994-95 | 22.6 | (17.9; 27.5) | 15.7 | (11.4; 20.9) | 10.3 | (6.5; 15.4) |
| 1996-97 | 19.7 | (15.8; 24.1) | 9.9 | (6.2; 13.9) | 19.7 | (12; 27.9) |
| 1998-99 | 18.2 | (13.6; 22.7) | 10.3 | (6.2; 14.8) | 11.8 | (6.7; 18.3) |
| 2000-01 | 24.5 | (19.5; 30.1) | 19.3 | (13.8; 25.3) | 11.7 | (6.1; 17.5) |
| 2002-03 | 19.6 | (14.6; 24.5) | 16.5 | (11.8; 22.3) | 12.3 | (7.4; 17.6) |
| 2004-05 | 20.4 | (14.6; 27.2) | 14.6 | (9.1; 21.7) | 10.7 | (5.7; 18.2) |
| 2006-07 | 17.5 | (12.6; 23.6) | 15.4 | (10.1; 22.2) | 13.5 | (6.9; 21.6) |
| 2008-09 | 16.0 | (10.5; 21.5) | 13.1 | (7.1; 21.4) | 11.1 | (4.6; 19) |
| 2010-11 | 12.9 | (7.5; 20.1) | 11.9 | (6.1; 20.5) | 10.4 | (3.4; 20.2) |
| 2012-13 | 13.0 | (6.7; 20.5) | 6.7 | (2.2; 12.5) | 6.0 | (1.6; 15.5) |
| Trend [*p*-value] | -0.86 | [<0.000] | -0.73 | [<0.000] | -0.69 | [<0.000] |
|  |  |  |  |  |  |  |
| **Island** | Luzon |  | Visayas |  | Mindanao |  |
| 1980-81 | 25.4 | (17.2; 34.1) | 29.1 | (16.3; 44.8) | 23.3 | (13.9; 34.6) |
| 1982-83 | 24.2 | (16.9; 33.8) | 27.5 | (17.3; 40) | 24.4 | (17; 33.6) |
| 1984-85 | 23.3 | (17.7; 29.8) | 31.8 | (21.6; 43.1) | 25.5 | (17.6; 34) |
| 1986-87 | 19.8 | (14.2; 25.6) | 19.8 | (13.2; 28.5) | 29.3 | (22.7; 38.3) |
| 1988-89 | 21.0 | (16.6; 26.4) | 22.2 | (15.6; 31.3) | 22.4 | (15.9; 29.1) |
| 1990-91 | 15.1 | (11.7; 19.1) | 21.7 | (14.8; 28.9) | 16.7 | (11.7; 22.3) |
| 1992-93 | 14.6 | (11; 18.6) | 17.4 | (11.8; 23.7) | 22.1 | (16.5; 28.1) |
| 1994-95 | 15.2 | (11.7; 19.1) | 21.5 | (14.6; 29.1) | 18.2 | (13.4; 24.1) |
| 1996-97 | 18.6 | (14.4; 22.8) | 12.4 | (8; 17.7) | 15.2 | (10.8; 20.2) |
| 1998-99 | 11.3 | (8.2; 15.5) | 21.2 | (14.5; 29.3) | 14.5 | (9.6; 19.2) |
| 2000-01 | 17.3 | (13.1; 21.4) | 24.5 | (16.8; 34.1) | 20.6 | (15.4; 26.8) |
| 2002-03 | 13.5 | (9.9; 17.5) | 26.1 | (17.2; 35.1) | 16.8 | (11.4; 23.7) |
| 2004-05 | 12.8 | (9; 17.8) | 28.4 | (18.7; 41.3) | 14.4 | (8.3; 21.8) |
| 2006-07 | 16.5 | (12.5; 22.5) | 15.4 | (7.5; 23.9) | 14.6 | (9.1; 21.4) |
| 2008-09 | 12.5 | (8.3; 19) | 14.9 | (6; 25.7) | 15.8 | (10.2; 23.1) |
| 2010-11 | 11.0 | (5.7; 16.3) | 9.1 | (2.3; 18.2) | 15.7 | (7.3; 26.2) |
| 2012-13 | 9.2 | (4.9; 15.6) | 6.8 | (0; 15.5) | 10.3 | (3.5; 18.7) |
| Trend [*p*-value] | -0.83 | [<0.000] | -0.93 | [0.005] | -0.82 | [<0.000] |

*Notes*: NMR, neonatal mortality rate; CI, confidence interval; Quin., Quintile. The small number of observations and possible non-linear relationships implies that the trend estimates should be treated with caution.
